# INTEGRATED DEMENTIA CARE CENTER IN TAIWAN: A SIX-YEAR JOURNEY TO DEVELOP A HOLISTIC CARE MODEL

**DOI:** 10.1093/geroni/igae098.2778

**Published:** 2024-12-31

**Authors:** Tsuann Kuo, Yi-Jia Huang, Yu-Chun Chen, Yu-Lan Lu, Yi-Hsiu Lin

**Affiliations:** Chung Shan Medical University, Taichung, Taichung, Taiwan (Republic of China); Chung Shan Medical University, Taichung, Taichung, Taiwan (Republic of China); Chung Shan Medical University Hospital, Taichung, Taichung, Taiwan (Republic of China); Chung Shan Medical University Hospital, Taichung, Taichung, Taiwan (Republic of China); Hsiang Shang Culture and Education Foundation, Taichung City, Taichung, Taiwan (Republic of China)

## Abstract

Taiwan launched its 8-year Dementia Action Plan 2.0 in 2017 and has developed over 114 Dementia Integrated Care Centers (DICC). Using the Chronic Disease Management Model, a multi-disciplinary and holistic care center was established by Chung Shan Medical University Hospital to care for more than 400 people with dementia and their family members each year. The DICC was consisted of medical, care, and support divisions so patients can be cared for by medical specialties, nurses, nutritionists, pharmacists, physical therapies, occupational therapies, social workers, psychologists and legal representatives. This study also analyzed over 2000 patients’ records retrospectively and found out that with the help of care managers at DICC, people with dementia were less likely to be hospitalized, entering emergency room, reduced caregiver burdens, and maintained cognitive level longer. The center also outreached to the community and established a network where people with dementia can attend physical, arts, leisure, and learning programs. The caregivers who were interviewed and attended these programs indicated that they were able to accept their family’s conditions faster and more at ease with their family dynamics. The DICC also served as an important site for University students to be trained to become future dementia specialists. In conclusion, a center with multi-disciplinary professionals took years to integrate and build collaborations so people with dementia and their families could get the best coordinated care. This paper will reveal how the Dementia Integrated Care Center will be implemented in Taiwan.

